# The JAM-B/c-src/MMP9 pathway is associated with progression and regulates the invasion of pancreatic cancer

**DOI:** 10.7150/jca.40953

**Published:** 2020-03-05

**Authors:** Wunai Zhang, Rui He, Shuo Chen, Li Zhang, Gang Cao, Wenbin Yang, Junhui Li

**Affiliations:** 1 Department of General Surgery, Second Affiliated Hospital, Xi'an Jiaotong University, 157 West 5 th Road, Xi'an, 710004, China.; 2 Department of Hepatobiliary Surgery, The First Affiliated Hospital of Xi'an Jiaotong University, Xi'an, 710061, China.; 3 Department of Oncology, The Second Affiliated Hospital of Xi'an Jiaotong University, 157 West 5 th Road, Xi'an, 710004, China.

**Keywords:** JAM-B, c-src, pancreatic cancer, progression, invasion

## Abstract

Junctional adhesion molecule B (JAM-B) is a multifunctional transmembrane protein that plays an important role in tumor progression. JAM-B is significantly upregulated in gastric cancer, melanoma cell metastasis and oral squamous cell carcinoma. JAM-2 may also function as a putative tumor suppressor in the progression and metastasis of colorectal cancer. The inconsistency of the results in different cancers has led to uncertainty regarding the role of JAM-B in carcinogenesis. For this purpose, the expression levels of JAM-B in pancreatic cancer (PanCa) tissues were associated with T stage and lymph node involvement with significant differences. A relatively high expression of JAM-B was found in PanCa cell lines by immunohistochemistry and western blot analysis. By cell transfection, JAM-B was silenced in tumor cell lines to determine cell invasion and migration abilities. Scratch wound assays and Transwell assays revealed that shJAM-B significantly decreased Panc-1 cell migration and invasion. Experiments were also conducted using a subcutaneous PanCa nude mouse model. A significant difference in tumor diameter at the injection site was found between the control group and the JAM-B low expression group. The expression levels of c-Src and MMP9 were also significantly reduced compared to that in the control group by immunohistochemistry. In conclusion, our results suggest that JAM-B secreted by cancer cells can promote progression and invasion in PanCa by upregulating the c-Src signal and related downstream proteins.

## Introduction

Pancreatic ductal adenocarcinoma (PanCa) is one of the most lethal human cancers, and complete surgical removal of the tumor followed by adjuvant chemotherapy is the only curative treatment 1. The estimated number of new cases of pancreatic cancer (PanCa) was 90,100, and 79,400 cases died in China in 2015. In the United States, the estimated number of new cases of PanCa was 55,440, and 44,330 cases died in 2018. PanCa ranks fourth among cancer-related deaths, and approximately 81% of patients present with regional and/or distant metastasis; from 2007 to 2013, the overall 5-year survival rate among patients with PanCa was <8% 2,3. Although research over the past decade has shown very interesting and promising new therapeutic options for these patients, only minor clinical success has been achieved.

The tumor microenvironment of PanCa is comprised of tumor cells, nontumor cells, extracellular matrix, cytokines, growth factors, and exosomes that regulate tumor progression and metastasis 4. Junctional adhesion molecules (JAMs) belong to the immunoglobulin (Ig) superfamily, and JAM-A, JAM-B, JAM-C, JAM-4, and JAM-like are family members 5. Junctional adhesion molecule B (JAM-B) is related to interendothelial cell-cell contacts, the formation of vascular tubes, the homeostasis of stem cell niches and the promotion of leukocyte adhesion and transmigration 6. JAM-B is also involved in the cellular motility and formation of cellular projections in cancer cells. JAM-B is highly expressed in gastric carcinoma, glioma and oral squamous cell carcinoma but is expressed at low levels in colorectal cancer and esophageal squamous cell carcinoma. The inconsistency of the results in different cancers has led to uncertainty regarding the role of JAM-B in carcinogenesis and metastasis 5.

As a member of the Src family of nonreceptor tyrosine kinases, c-Src is often upregulated in a variety of human tumors. c-Src functions as a critical link between multiple signaling pathways that regulate proliferation, invasion, survival, metastasis and angiogenesis 7. JAM-B and JAM-C could activate the c-Src proto-oncogene, which is known as a central upstream molecule in the pathways regulating cell invasion and migration. The relationship between JAM-B and c-Src is unclear 8.

In this study, we explored the role of JAM-B in the progression and metastasis of PanCa and its potential signaling mechanism.

## Materials and methods

### Tissue samples

A total of 712 pancreatic tumors were subjected to clinical examination at the Second Affiliated Hospital of Xi'an Jiaotong University, China. The Inclusion criteria include: 1) the pathological diagnosis is pancreatic adenocarcinoma through EUS or CT guided puncture biopsy, cytological examination of ascites exfoliation or exploratory biopsy under laparoscopy or laparotomy; 2) no age or gender limit; 3) no neoadjuvant therapy was received before surgery. The exclusion criteria include: 1) Patients with more than two kinds of tumors at the diagnosis time; 2) fewer than 15 lymph nodes were dissected during the surgery. Among them, 105 patients who underwent pancreatic surgery had a confirmed pathological diagnosis of pancreatic ductal carcinoma. The median patient age was 61.5 ±11.5 years, and 42 of the patients were female. None of the 105 patients received neoadjuvant therapy before their operation. As summarized in Table 1, four clinical parameters were evaluated in each group, including T stage, stage grouping, lymph node involvement and metastases.

### Immunohistochemistry (IHC)

Immunohistochemical staining was performed on 3 mm sections of the formalin-fixed paraffin-embedded samples using a standard avidin-biotin complex peroxidase technique. After deparaffinization with xylene and rehydration with serial gradient ethanol, the antigen was retrieved by heating the slides in 10 mM of citrate buffer (pH 6.0) for 20 min in a microwave. The endogenous peroxidase was blocked with 0.3% hydrogen peroxide. The slides were subsequently incubated with a blocking protein (Dako, CA, USA) for 10 min, and primary antibody was added overnight at 4°C followed by washing. The following antibodies were used: monoclonal anti-JAM-B (Santa Cruz Biotechnology Co., Ltd., Shanghai, China), monoclonal anti-JAM-C (Santa Cruz Biotechnology Co., Ltd., Shanghai, China), monoclonal anti-c-src (Santa Cruz Biotechnology Co., Ltd., Shanghai, China), monoclonal anti-MMP2 (Santa Cruz Biotechnology Co., Ltd., Shanghai, China), and monoclonal anti-MMP9 (Santa Cruz Biotechnology Co., Ltd., Shanghai, China). The secondary biotinylated goat anti-rabbit antibody or goat anti-mouse antibody (Dako) was then applied for 30 min, followed by 30 min of incubation with streptavidin peroxidase (Dako LSAB+ HRP kit). After washing, the slides were visualized by diaminobenzidine (DAB) chromogen solution (Dako) and counterstained with routine hematoxylin, followed by dehydration through graded ethanol and mounting of the slides. The control group was selected from ten normal pancreatic tissues from euglycemic patients with normal body mass indices and whose ages had been matched with those of the patients in the experimental groups. To ensure the specificity of the primary antibodies, consecutive tissue sections were incubated in the absence of the primary antibody. No immunostaining was detected in these sections, showing the specificity of the primary antibodies used in this study.

### Cells and cell culture

PanCa cell lines, including Panc-1, BxPC-3, AsPc-1, CFPAC-1, and SW1990, were obtained from the American Type Culture Collection (ATCC) and maintained in Dulbecco's modified Eagle's medium (DMEM; HyClone), RPMI-1640 (Thermo Fisher Scientific Inc., Shanghai, China) or Leibovitz's L15 Medium (Sigma-Aldrich, Inc.) supplemented with 10% heat-inactivated fetal bovine serum (FBS, Sigma-Aldrich, Inc.), 100 μg/ml penicillin G and 100 μg/ml streptomycin sulfate (Sigma-Aldrich) in an incubator at 37.0°C with 95% humidity and 5% CO2. The cell lines were obtained directly from the ATCC, which conducts cell line characterizations or authentication by short tandem repeat profiling, and passaged in our laboratory for less than 6 months after receipt.

### Plasmid construction and transfections

Cells were seeded into small dishes and transfected with shRNA using Lipofectamine Transfection Reagent (Invitrogen, CA, USA) according to the manufacturer's instructions. The cells were used for further experiments 24 h after transfection. The JAM-2-GFP and pCMV-entry plasmids were purchased from OriGene Technologies, Inc. (Rockville, MD). The JAM-2 cDNA sequences obtained from the JAM-2-GFP plasmid were individually cloned into a mammalian expression pCMV-entry plasmid vector. Purified JAM-2 transgenes and control plasmid vectors were transfected into Panc-1 and BxPC-3 cells using an Easyjet Plus electroporator (EquiBio Ltd., Kent, UK). One day before transfection, the cells were plated in the appropriate amount of growth medium without antibiotics such that they would be 30% confluent at the time of transfection. The DNA and RNAi molecules were diluted in the appropriate amount of Opti-MEM I Medium (Thermo Fisher Scientific Inc., Shanghai, China) without serum. Lipofectamine 2000 (Thermo Fisher Scientific Inc., Shanghai, China) was mixed gently before use and then diluted in Opti-MEM I Medium without serum. This solution was mixed gently and incubated for 5 min at room temperature. After the 5-minute incubation, the diluted DNA and RNAi molecules were combined with the diluted Lipofectamine 2000. This solution was mixed gently and incubated for 15 min at room temperature to allow complex formation to occur. Although the solution may appear cloudy, this will not impede the transfection. The DNA-shRNA molecule-Lipofectamine 2000 complexes were added to each well containing the cells and medium. Then, the plate was mixed gently by rocking. The cells were incubated at 37 °C in a CO2 incubator until they were ready to be harvested and assayed for the target gene. The removal of complexes or media change was not required; however, the growth medium could be replaced after 4-6 h without loss of transfection activity.

### Cell scratch wound assay

JAM-B cells (700,000/well) were seeded in a 24-well plate and cultured in the incubator overnight. Then, the cultured cells were scratched with a 200-µl pipette tip to create a wound and washed twice with phosphate-buffered saline (PBS) to remove floating cells. The cells were photographed at intervals using an inverted microscope. The size of the wounds was subsequently measured with ImageJ software.

### *In vitro* cell invasion assay

Transwell inserts with an 8 µm pore size were coated with 50 µl of Matrigel (BD Matrigel™ Basement Membrane Matrix) and air-dried. Cell suspensions (shJAM-B, NSC and control) were added to the upper chambers in medium containing 10% FBS up to confluence and replaced with 0.5% FBS. Following rehydration, 400 µl/well medium was added to the bottom chambers with 30% FCs. After 24 h, the noninvading cells were removed from the bottom surface by scraping with a wet cotton swab. After rinsing with PBS, the filter was fixed with 4% formalin and stained with crystal violet. The invasion ability was determined by counting the stained cells on the bottom surface.

### Western blot analysis

Total protein was extracted from cultured PanCa cells in cell lysis buffer with protease inhibitors (Roche, Penzberg, Germany) on ice for 20 min. Equal amounts of protein sample were separated using 10% sodium dodecyl sulfate-polyacrylamide gel electrophoresis (SDS-PAGE) and blotted onto PVDF membranes. The membranes were incubated with a blocking buffer for 15 min. Primary antibodies against JAM-B, JAM-C, c-Src, MMP2, and MMP9 (Santa Cruz) and a secondary antibody (anti-rabbit IgG or anti-mouse IgG; Santa Cruz) were used. Equal protein sample loading was monitored using an anti-b-actin antibody (Santa Cruz). Protein bands were visualized and analyzed using an enhanced chemiluminescence detection system (Amer sham Biosciences) and transferred onto X-ray film. Athymic nude mice were purchased from the Shanghai Laboratory Animal Center.

### Ectopic tumor model

The shJAM-B and NSC cells were prepared as described for the subcutaneous injection model. A total of 30 μl of the cell suspension (concentration of 0.5x10^6^/μL) was subcutaneously injected into the flank of 8- to 10-week-old female athymic nude mice. The sites were examined daily to determine whether tumors had appeared; after tumors appeared, their sizes were measured weekly. After 3 weeks, the mice were sacrificed, and the tumors were harvested for the evaluation of tumor growth and diameter. Immunohistochemical staining was performed on 3 mm sections of the formalin-fixed paraffin-embedded samples using a standard avidin-biotin complex peroxidase technique. The animal care and experiments conformed to the Declaration of Helsinki and were approved by the Ethical Review Board (ERB) Committee of the Second Affiliated Hospital, Xi'an Jiaotong University, China.

### Statistical analysis

The analyses of results were performed using the SPSS statistical software package (version 18.0; SPSS Inc.). The significance of the data was determined using Student's t test or one-way analysis of variance (ANOVA) for the *in vitro* and *in vivo* results. The significance level was set at P < 0.05. All results are expressed as the means ± SDs. All experiments were repeated 3 times, with n = 6 per group.

## Results

### Expression of JAM-B in pancreatic cancer (PanCa) tissues and its association with patient progression

The histopathological characteristics of 712 PanCa cases obtained from the Second Affiliated Hospital of Xi'an Jiaotong University used in this study are presented in Table 1. Ultimately, 105 patients who underwent pancreatic surgery had a confirmed pathological diagnosis of pancreatic ductal carcinoma. The median patient age was 61.5 ±11.5 years, and 42 of the patients were female. None of the 105 patients received neoadjuvant therapy before their operation.

**Figure 1:**
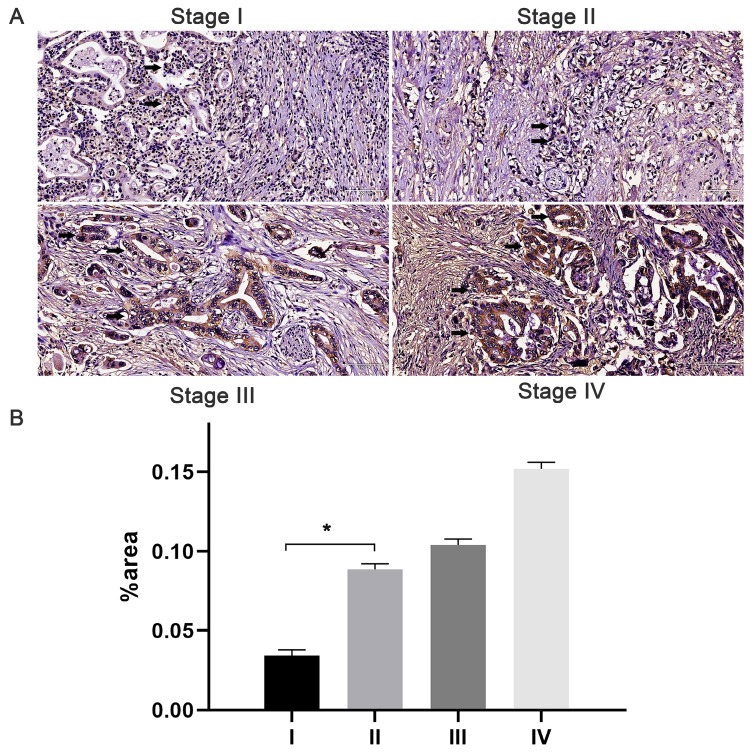
Expression of JAM-B in pancreatic cancer (PanCa) tissues and its association with patient progression. (A) Expression of JAM-B in stage I, II, III and IV PanCa. Arrowheads show the cells of interest. (B) Relative protein levels of JAM-B are shown as bar diagrams (*P<0.05, compared with stage I).

The immunostaining of JAM-B was localized to the cytoplasm and membrane of pancreatic tissues. With the increase in T stage, comparable increases of positive immunostaining were present (Fig. 1). Of the 105 cases, 84 (80%) had JAM-B positive expression, and the cases were then divided into Groups 1 (JAM-B positive) and 2 (JAM-B negative). As shown in Table 1, the median age was 63.7 ±12.1 and 57.6±10.4 years, and 50 and 13 of the patients were male in Group 1 and Group 2, respectively, with no significant difference in these 2 groups.

The frequencies of T1 stage were 3.57% (3/84) and 33.33% (7/21) in Group 1 and Group 2, respectively, with significant differences between the two groups (P = 0.000, Chi-square test). The frequencies of T2 stage were 28.57% (24/84) and 52.38% (11/21) in Group 1 and Group 2, respectively, with significant differences between the two groups (P = 0.016, Chi-square test). The frequencies of T3 stage were 59.52% (50/84) and 9.52% (2/21) in Group 1 and Group 2, respectively, with significant differences between the two groups (P =0.000, Chi-square test). The frequencies of T4 stage were 8.33% (7/84) and 4.76% (1/21) in Group 1 and Group 2, respectively, with no significant difference between the two groups (P = 0.927, Chi-square test). We also found that the percentage of patients in stage I (24.42%, P=0.008) was lower in the positive group than that in the negative group (63.16%) and that the percentage of patients in stage II (63.95%, P=0.003) in the JAM-B positive group was lower than that in the negative group (31.57%). We then tested lymph node involvement and found that 64.29% (54/84) of patients in Group 1 and 28.57% (6/21) in group 2 had N1 lymph node involvement with a significant difference between the two groups (P =0.006, Chi-square test). Three cases had M1 metastasis in Group 1, and none had M1 metastasis in Group 2, with no significant difference (P = 0.884, Chi-square test).

### Expression of JAM-B in PanCa cell lines and the effect of silencing JAM-B on the cell signaling pathway

**Figure 2:**
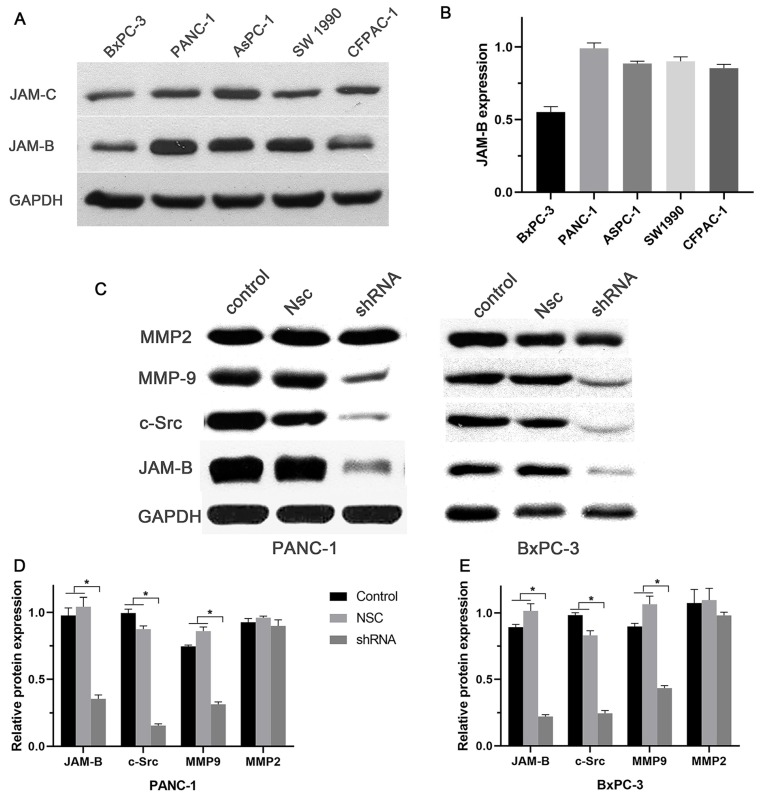
Expression of JAM-B in pancreatic cancer (PanCa) cell lines, and the effect of the silencing JAM-B on cell signal pathway. (A)Western blot was used to detect the protein level of JAM-B and JAM-C in 5 PanCa (BxPC-3, Panc-1, AsPC-1, SW1990 and CFPAC-1 cell lines. (B) Western blot analysis with IMAGE J was used to detect the protein level of JAM-B and JAM-C in 5 PanCa cells. The expression of each protein in cells was determined following normalization with a loading control, GAPDH. (C) Transfection efficiency of JAM-B in PanCa cell lines following transfection with JAM-B shRNA at the protein level determined by western blot analysis and the downstream pathway was also analyzed in BxPC-3 and Panc-1. (D and E) Western blot analysis with IMAGE J was used to detect the protein level of JAM-B and the downstream pathway. The expression of each protein in cells was determined following normalization with a loading control, GAPDH. Relative protein levels of JAM-B are shown as bar diagrams (*P<0.05, compared with control and NSC).

Our *in vitro* results showed a relatively high expression of JAM-B and low expression of JAM-C in the 5 pancreatic cancer cell lines (Fig. 2A and B). We then chose Panc-1 and BxPC-3 for the next experiment. To study the role of JAM-B in PanCa metastasis and progression, the downregulation of JAM-B was performed, and empty JAM-B plasmids (Nsc) and nontransfected cells were used as controls. Once the Panc-1 and BxPC-3 cells were transfected with sh-JAM-B, an evaluation with western blotting and densitometry was performed. As shown in Fig. 2C, the expression of JAM-B was lower than that in the NSC and control groups. The expression of c-src and MMP9 was also lower than that in the Nsc and control groups. Densitometry showed that JAM-B was significantly reduced in Panc-1 and BxPC-3 cells when transfected with sh-JAM-B, and these cells exhibited a decrease in JAM-B expression compared to that in cells that were not transfected and NSCs with significant differences (Fig. 2D and E, P<0.05). We also compared the expression of c-src, MMP2 and MMP9 and found that the expression of c-src and MMP9 were also reduced in the transfected group compared to the NSC and control groups, with significant differences (Fig. 2D and E, P<0.05). The expression of MMP2 was not significantly different in the 3 groups.

### Effects of silencing JAM-B on cell migration in PanCa cell lines

**Figure 3:**
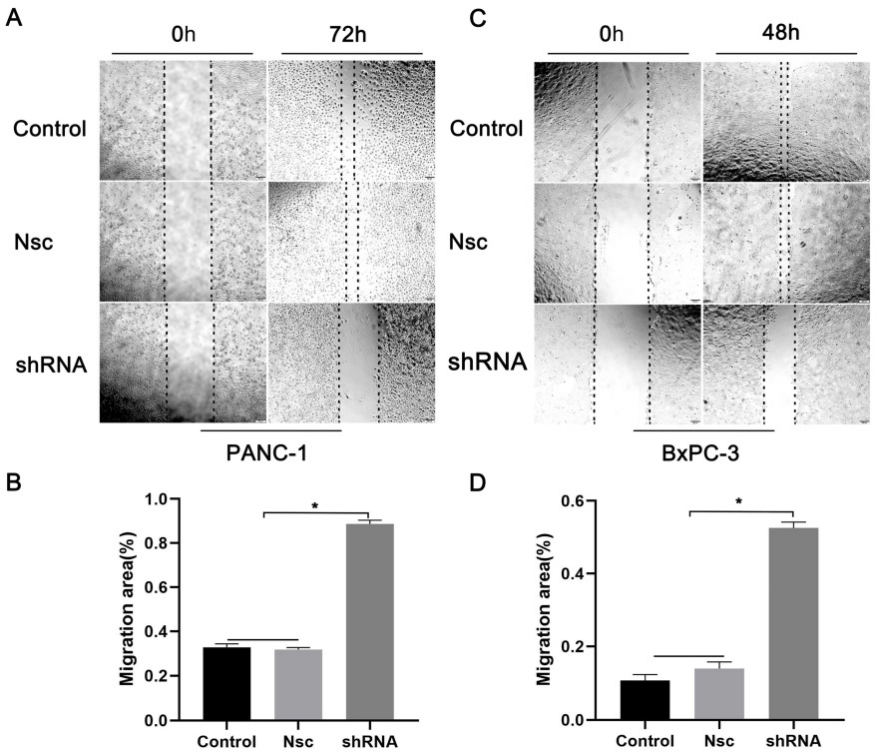
Effects of silencing JAM-B on cell migration in PanCa cell lines. (A) Cell scratch wound assay was carried out to detect the effects of the silencing of JAM-B on the migration of PANC-1at 0h and 72h. (B) Relative migration levels of PANC-1 are shown as bar diagrams (*P<0.05, compared with control and Nsc). (C) Cell scratch wound assay was carried out to detect the effects of the silencing of JAM-B on the migration of BxPC-3 at 0h and 48h. (D) Relative migration levels of BxPC-3 are shown as bar diagrams (*P<0.05, compared with control and Nsc).

We examined the effects of JAM-B on PanCa cell migration and invasion. As shown in Fig. 3A, shJAM-B significantly decreased Panc-1 cell migration at 72 h by the method of wound healing assays. We then calculated the migration area and found that shJAM-B significantly decreased Panc-1 cell migration compared to that of the Nsc and control groups (Fig. 3B, P<0.05). The shJAM-B also significantly decreased BxPC-3 cell migration at 48 h compared to that of the Nsc and control groups (Fig. 3C and D, P<0.05).

### Effects of silencing JAM-B on cell invasion in PanCa cell lines

**Figure 4:**
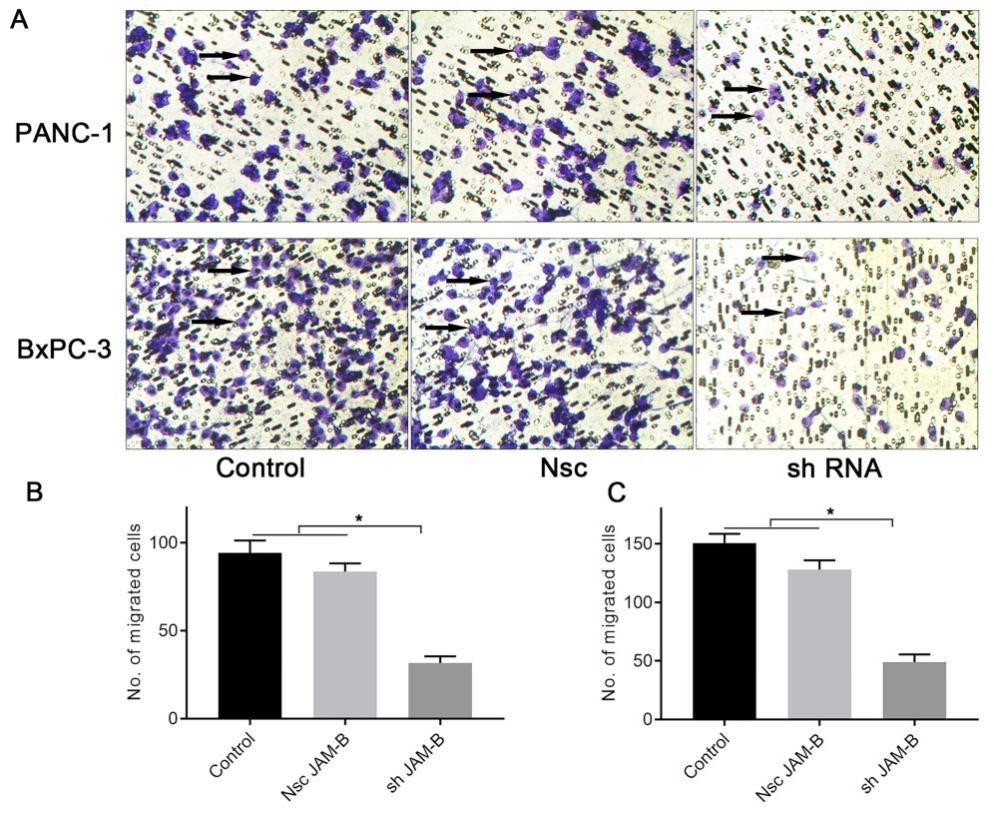
Effects of silencing JAM-B on cell invasion in PanCa cell lines. (A) Transwell assay was performed to detect the effects of the silencing of JAM-B on invasion of PANC-1 and BxPC-3. Arrowheads show the cells of interest. (B) Relative invasion cells are shown as bar diagrams in PANC-1. (C) Relative invasion cells are shown as bar diagrams in BxPC-3. Data are shown as the means ± SD from 3 independent experiments. (*P<0.05, compared with control and Nsc).

We then examined the effects of JAM-B on PanCa cell invasion by the method of Transwell assays. As shown in Fig. 4A, shJAM-B significantly decreased Panc-1and BxPC-3 cell invasion at 24 h. We then calculated the positive cells and found that shJAM-B significantly decreased Panc-1 and BxPC-3 cell invasion compared to that of the Nsc and control groups (Fig. 4B and C, P<0.05).

### Effects of silencing JAM-B on cell invasion in a PanCa subcutaneous nude mice model

**Figure 5:**
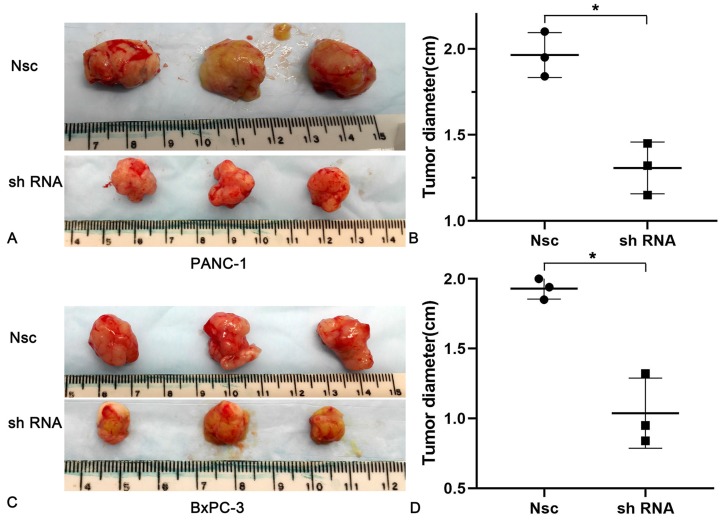
Effects of silencing JAM-B on cell invasion in PanCa subcutaneous nude mice model. (A and B) The tumor diameters increased more slowly in the JAM-B low expression groups than that in Nsc group in PANC-1. (C and D) The tumor diameters increased more slowly in the JAM-B low expression groups than that in Nsc group in BxPC-3. (*P<0.05, compared with NSC).

To better understand the impacts of JAM-B on PanCa, nude mice (n = 6 per group) were subcutaneously injected with 5 × 10^5^ PanCa cells (NSC and shJAM-B). Two weeks later, all mice showed tumor formation in the JAM-B+ and JAM-B- groups. Compared with that in the control group, the tumor diameters increased more slowly in the JAM-B low expression groups. At the end of the experiment (3 weeks after injection), all mice were sacrificed for histological examinations of the tumor tissues (Fig. 5A and C). We found a significant difference in tumor diameter at the injection site between the control group and the JAM-B low expression group (Fig. 5B and D).

**Figure 6:**
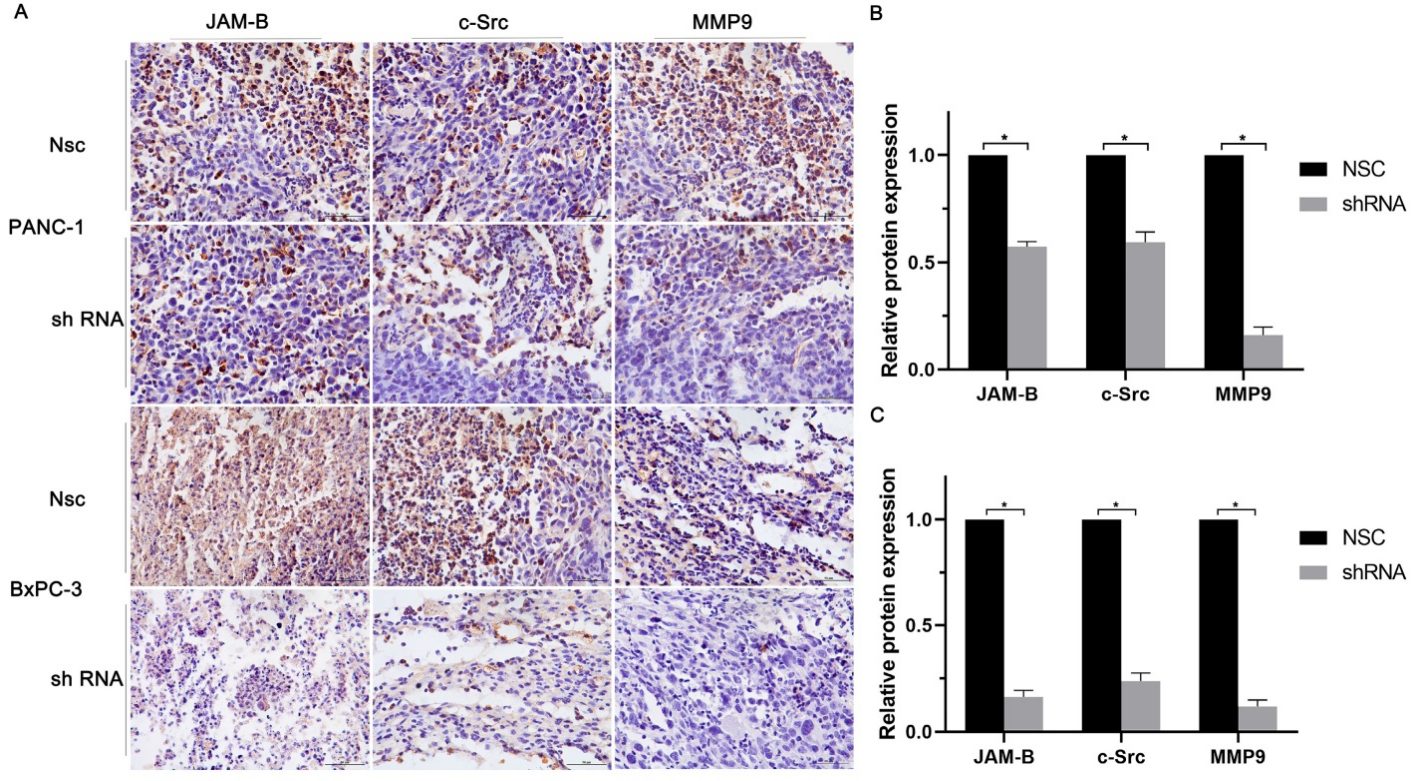
Relationship of JAM-B-c-src-MMP9 axis and pancreatic cancer progression *in vivo*. (A) Immunohistochemical staining of JAM-B, c-src and MMP9 were used to detect the protein level of PANC-1 and BxPC-3. (B and C) Immunohistochemical staining analysis with IMAGE J was used to detect the protein level of JAM-B and the downstream pathway of PANC-1(B) and BxPC-3(C). Relative protein levels of JAM-B are shown as bar diagrams (*P<0.05, compared with control and Nsc).

We then quantified the expression of JAM-B, c-src, and MMP9 in solid tumor paraffin sections by immunohistochemistry and found that the expression of JAM-B, c-src and MMP9 were significantly reduced compared to those in the control group by immunohistochemistry (Fig. 6A). We then calculated the relative expression of JAM-B, c-src and MMP9 and found that shJAM-B significantly decreased the expression of the above proteins compared to that of the Nsc groups (Fig. 6B and C, P<0.05).

## Discussion

In contrast to declining trends for the major cancers, a join point analysis indicated that from 1930 to 2016, the death rates rose in both sexes for pancreatic cancers 9. PanCa is estimated to be the leading cause of cancer-related deaths in the USA by 2050(9). Therefore, to provide optimal care for patients with PanCa, we need to better understand its complex molecular biology and thus to identify new target molecules that target the proliferation and invasion of PanCa cells.

JAM-B is a multifunctional transmembrane protein that belongs to the immunoglobulin superfamily. JAM-B plays an important role in numerous cellular processes, such as tight junction assembly, spermatogenesis, and regulation of paracellular permeability, leukocytic transmigration, angiogenesis, cell proliferation and tumor metastasis 5. Hajjari et al found that JAM-B was upregulated significantly in gastric tumor samples compared with adjacent normal tissues, and its expression was higher in high grade tumors than in low grade and intermediate grade tumors, which promoted the progression of cancer by regulating the expression level of the actin filament-associated protein gene that appears to be a downstream 10. Our results showed that 80% of PanCa samples had JAM-B positive expression, which is related to high grade, tumor stage and lymph node involvement. Thus, JAM-B may promote cancer progression by some unclear mechanisms.

Using the method of whole genome and transcriptome sequencing in primary and peritoneal metastatic gastric carcinoma, JAM-B was found to be mutated in primary gastric tumors 11. JAM-B was also involved in melanoma cell metastasis via its interaction with JAM-C on tumor cells. As a counter-receptor for JAM-C, JAM-B also mediated adhesion to primary lung microvascular endothelial cells and the reservation of melanoma cells in the lungs 12. JAM-B was upregulated in human oral cancer LNM Tca8113 cells that metastasized to lymph nodes at a higher rate than its parental cell line Tca8113, indicating that JAM-B acts as a potential factor for the metastasis of oral squamous cell carcinoma (OSCC) 13.

JAM-2 may function as a putative tumor suppressor in the progression and metastasis of colorectal cancer 14. The inconsistency of the results in different cancers has led to an uncertainty regarding the role of JAM-B in carcinogenesis 15. Kok-Sin et al reported that JAM-B was expressed at very low levels in colorectal cancer due to JAM-B genes being hypermethylated on promoters in CpG islands with methylation-specific multi-plex ligation-dependent probe amplification (MS-MLPA) 14. Bujko et al also found that JAM-B was expressed at lower levels in adenocarcinoma and adenoma than in normal colonic mucosa. Furthermore, JAM-B had the lowest expression levels in adenoma 16. Li et al also demonstrated that the methylation of JAM-B was increased in the region downstream of the gene and that its expression was decreased 17.

The inconsistency of the results in different cancers has led to uncertainty regarding the role of JAM-B in carcinogenesis; thus, the aim of this study was to explore the potential role of JAM-B in PanCa. Our results showed abnormally high expression levels of JAM-B in pancreatic tumor cells. To study the role of JAM-B in PanCa metastasis and progression, wound healing and Transwell assays were used. We observed that the migratory and invasive abilities of shJAM-B Panc-1 and BxPC-3 cells were significantly reduced compared with those of the NSC and control cells.

C-Src is a member of the Src family of nonreceptor tyrosine kinases and functions as a critical link between multiple signaling pathways that regulate proliferation, invasion, survival, metastasis and angiogenesis. The molecular-targeted therapy of c-Src has thus emerged as a promising treatment for cancer 7,18,19. C-Src activity may directly regulate BGC-823 cell invasion and migration by modulating the maturation of MT1-MMP and VEGF-C. The invasive ability can be impaired by c-Src inhibitors in correlation with downregulated MMP2 and MMP9 activity 20.

The interaction of JAM-B and JAM-C could activate the c-Src proto-oncogene, which is known as a central upstream molecule in the pathways regulating cell invasion and migration 8. The regulation of the tumor suppressor protein E-cadherin plays an important role in cancer development and progression and may contribute to the decision between 'single cell' and 'collective invasion' *in vivo*. Mounting evidence from *in vitro* and *in vivo* experiments place nonreceptor protein tyrosine kinases, Src and focal adhesion kinase (FAK); at the heart of e-cadherin regulation and the crosstalk between integrins and cadherins 21. Canel et al investigated the role of Src/FAK signaling in E-cadherin and cell-cell adhesion regulation and implied that the inhibition of this signaling pathway strengthens cell-cell adhesion *in vivo*, promotes a more epithelial-like morphology and suppresses epithelial tumor cell migration 22. We further showed that PanCa cells overexpressing JAM-B displayed significantly increased c-Src, E-Cadherin, MMP-2 and MMP-9, which indicated that c-Src signaling may be involved in the progression of PanCa. Our *in vivo* tumorigenicity model also showed the effects of JAM-B on promoting tumor incidence, the time to tumor appearance, tumor size, tumor weight, and the expression of related factors such as c-Src, E-Cadherin, MMP-2 and MMP-9, which provided necessary support for future mechanistic studies. Our *in vivo* experiments produced similar results.

In conclusion, the previous study showed that the interaction of JAM-B and JAM-C could activate the c-Src proto-oncogene, which regulate cell invasion and migration. Our results showed that JAM-B can activate the c-Src/MMP9 pathway directly, has important clinical implications because it involves novel pharmacologic targets that may be targeted to reduce tumor neuroinvasion and to improve the survival of patients with PanCa.

## Figures and Tables

**Table 1:** Clinical parameters for JAM-B+/- cases

Variable	Patients	Positive	Negative	P-value
Age (years)	61.5 ±11.5	63.7 ±12.1	57.6±10.4	
Male/Female	63/42	50/34	13/8	**1.0**
T stage				
T1	10	3	7	0.000*
T2	35	24	11	0.016*
T3	52	50	2	0.000*
T4	8	7	1	**0.927**
Stage Grouping				
0	0	0	0	
I	33	21	12	0.008*
II	61	55	6	0.003*
III	8	7	1	**0.927**
IV	3	3	0	**0.884**
Lymph node				
N0	45	30	15	
N1	60	54	6	0.006*
Metastases				
M0	102	81	21	
M1	3	3	0	**0.884**

*indicates P-value <0.05.

## References

[1] Birnbaum DJ, Bertucci F, Finetti P (2018). Molecular classification as prognostic factor and guide for treatment decision of pancreatic cancer. Biochim Biophys Acta Rev Cancer.

[2] Chen W, Zheng R, Baade PD (2016). Cancer statistics in China, 2015. CA Cancer J Clin.

[3] Siegel RL, Miller KD, Jemal A (2018). Cancer statistics, 2018. CA Cancer J Clin.

[4] Ansari D, Friess H, Bauden M (2018). Pancreatic cancer: disease dynamics, tumor biology and the role of the microenvironment. Oncotarget.

[5] Zhao H, Yu H, Martin TA (2016). The role of JAM-B in cancer and cancer metastasis (Review). Oncol Rep.

[6] Stelzer IA, Mori M, DeMayo F (2016). Differential mouse-strain specific expression of Junctional Adhesion Molecule (JAM)-B in placental structures. Cell Adh Migr.

[7] Okamoto W, Okamoto I, Yoshida T (2010). Identification of c-Src as a potential therapeutic target for gastric cancer and of MET activation as a cause of resistance to c-Src inhibition. Mol Cancer Ther.

[8] Tenan M, Aurrand-Lions M, Widmer V (2010). Cooperative expression of junctional adhesion molecule-C and -B supports growth and invasion of glioma. Glia.

[9] Siegel RL, Miller KD, Jemal A (2019). Cancer statistics, 2019. CA Cancer J Clin.

[10] Hajjari M, Behmanesh M, Sadeghizadeh M (2013). Junctional adhesion molecules 2 and 3 may potentially be involved in progression of gastric adenocarcinoma tumors. Med Oncol.

[11] Zhang J, Huang JY, Chen YN (2015). Whole genome and transcriptome sequencing of matched primary and peritoneal metastatic gastric carcinoma. Sci Rep.

[12] Arcangeli ML, Frontera V, Bardin F (2012). The Junctional Adhesion Molecule-B regulates JAM-C-dependent melanoma cell metastasis. FEBS Lett.

[13] Zhuang Z, Jian P, Longjiang L (2010). Oral cancer cells with different potential of lymphatic metastasis displayed distinct biologic behaviors and gene expression profiles. J Oral Pathol Med.

[14] Kok-Sin T, Mokhtar NM, Ali Hassan NZ (2015). Identification of diagnostic markers in colorectal cancer via integrative epigenomics and genomics data. Oncol Rep.

[15] Gungor C, Hofmann BT, Wolters-Eisfeld G (2014). Pancreatic cancer. Br J Pharmacol.

[16] Bujko M, Kober P, Mikula M (2015). Expression changes of cell-cell adhesion-related genes in colorectal tumors. Oncol Lett.

[17] Li X, Wu Z, Mei Q (2013). Long non-coding RNA HOTAIR, a driver of malignancy, predicts negative prognosis and exhibits oncogenic activity in oesophageal squamous cell carcinoma. Br J Cancer.

[18] Nam HJ, Im SA, Oh DY (2013). Antitumor activity of saracatinib (AZD0530), a c-Src/Abl kinase inhibitor, alone or in combination with chemotherapeutic agents in gastric cancer. Mol Cancer Ther.

[19] Summy JM, Gallick GE (2006). Treatment for advanced tumors: SRC reclaims center stage. Clin Cancer Res.

[20] Yang Y, Bai ZG, Yin J (2014). Role of c-Src activity in the regulation of gastric cancer cell migration. Oncol Rep.

[21] Serrels A, Canel M, Brunton VG (2011). Src/FAK-mediated regulation of E-cadherin as a mechanism for controlling collective cell movement: insights from *in vivo* imaging. Cell Adh Migr.

[22] Canel M, Serrels A, Miller D (2010). Quantitative *in vivo* imaging of the effects of inhibiting integrin signaling via Src and FAK on cancer cell movement: effects on E-cadherin dynamics. Cancer Res.

